# Supplementary material for: A humanized anaplastic lymphoma kinase (ALK)-directed antibody-drug conjugate with pyrrolobenzodiazepine payload demonstrates efficacy in ALK-expressing cancers
Source: Nat Commun. 2025 Aug 15;16:7578. doi: 10.1038/s41467-025-62979-1 (PMC12354906; doi:10.1038/s41467-025-62979-1)
Supplement: Supplementary file 1 — Supplementary Information [file 41467_2025_62979_MOESM1_ESM.pdf]

Supplemental Figure 1

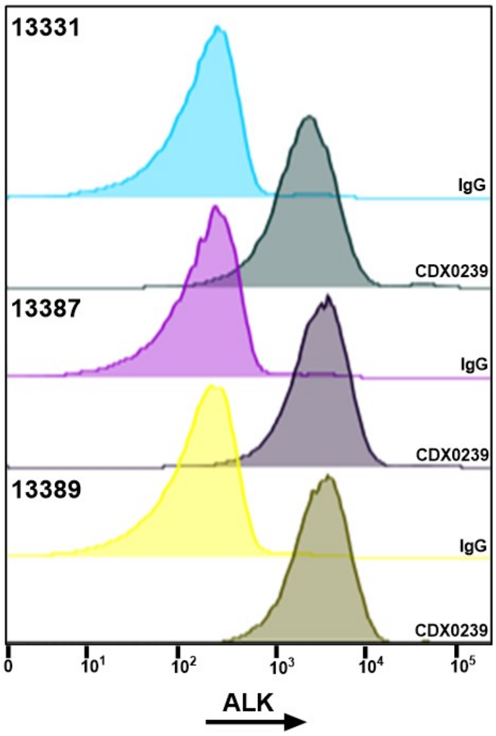

Supplemental Figure 1. CDX0239 binds to cell surface ALK in spontaneous murine neuroblastoma tumors. Flow cytometry histograms for ALK using CDX0239 compared to IgG control in spontaneously occurring murine neuroblastoma tumors 13331, 13387, and 13389 derived from Th-MYCN models. Represented data has been validated with at least 3 independent experiments from different biological samples derived from the spontaneous tumors of individual mice.

Supplemental Figure 2

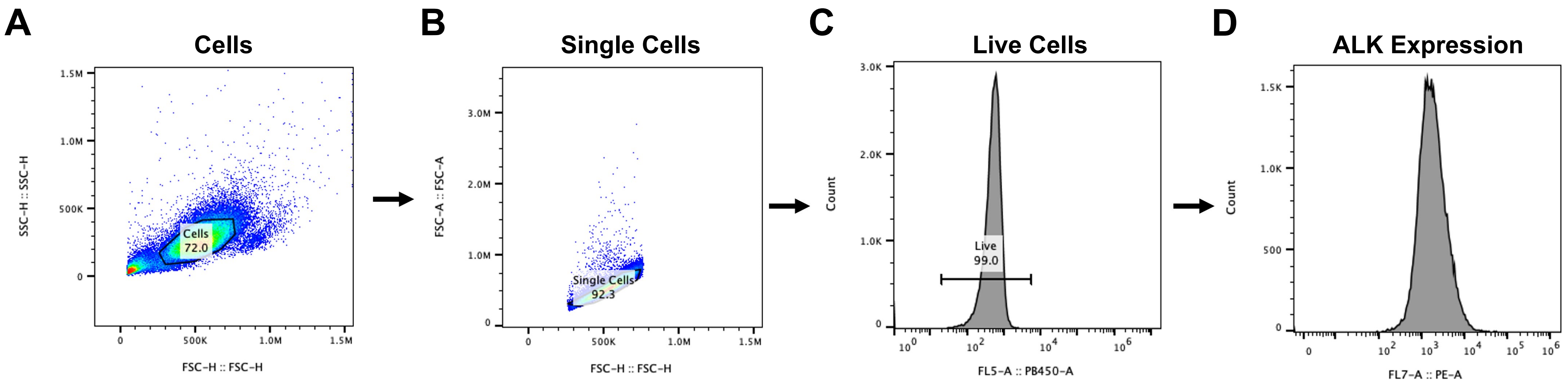

Supplemental Figure 2. Gating strategy for flow cytometry analysis for ALK surface expression after mouse cell depletion. (A) Side scatter height vs forward scatter height plot for gating removal of debris. **(B)** Forward scatter area vs forward scatter height plot for gating of single cells. **(C)** PB450-A positive cell population histogram for analysis of live cells. **(D)** PE positive cell population histogram for analysis of ALK cell surface expression.

Supplemental Figure 3

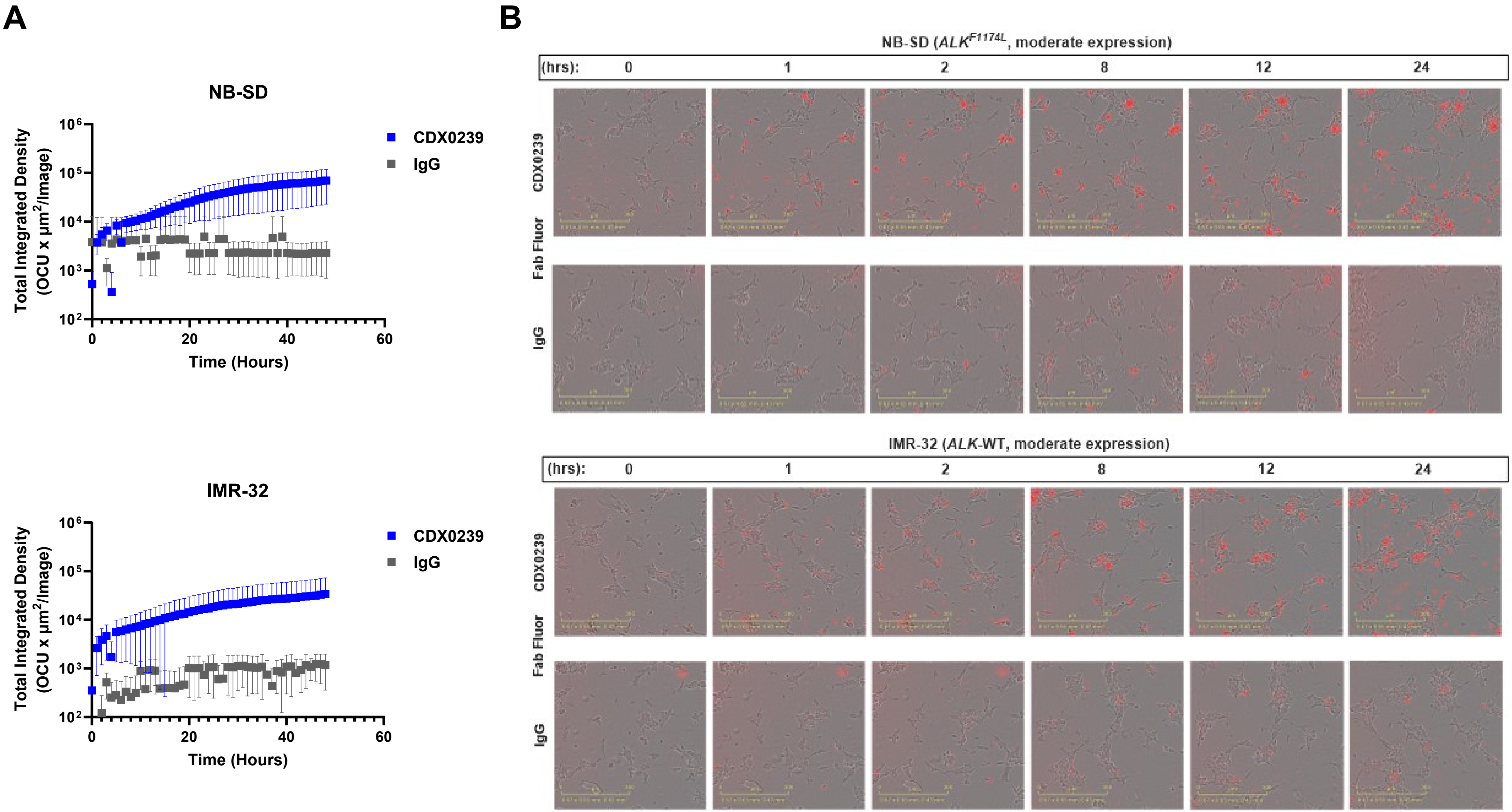

Supplemental Figure 3. CDX0239 is internalized in moderate surface ALK-expressing neuroblastoma cell lines. (A) Quantification of internalized Fabfluor-conjugated CDX0239 (blue line with squares) compared to Fabfluor-conjugated IgG control (grey line with squares) in NB-SD and IMR-32 cell lines plotted as total integrated density mean ± standard deviation over 48 hours with n=3 technical replicates for the CDX0239 condition in NB-SD and n=6 technical replicates for all other conditions. Sample size and datasets are located within the Source Data File. Source data are provided as a Source Data File. **(B)** Pictographs of the internalization of Fabfluor-conjugated CDX0239 compared to Fabfluor-conjugated IgG in NB-SD and IMR-32 cell lines over 24 hours and represented data has been validated with at least 2 independent experiments.

Supplemental Figure 4

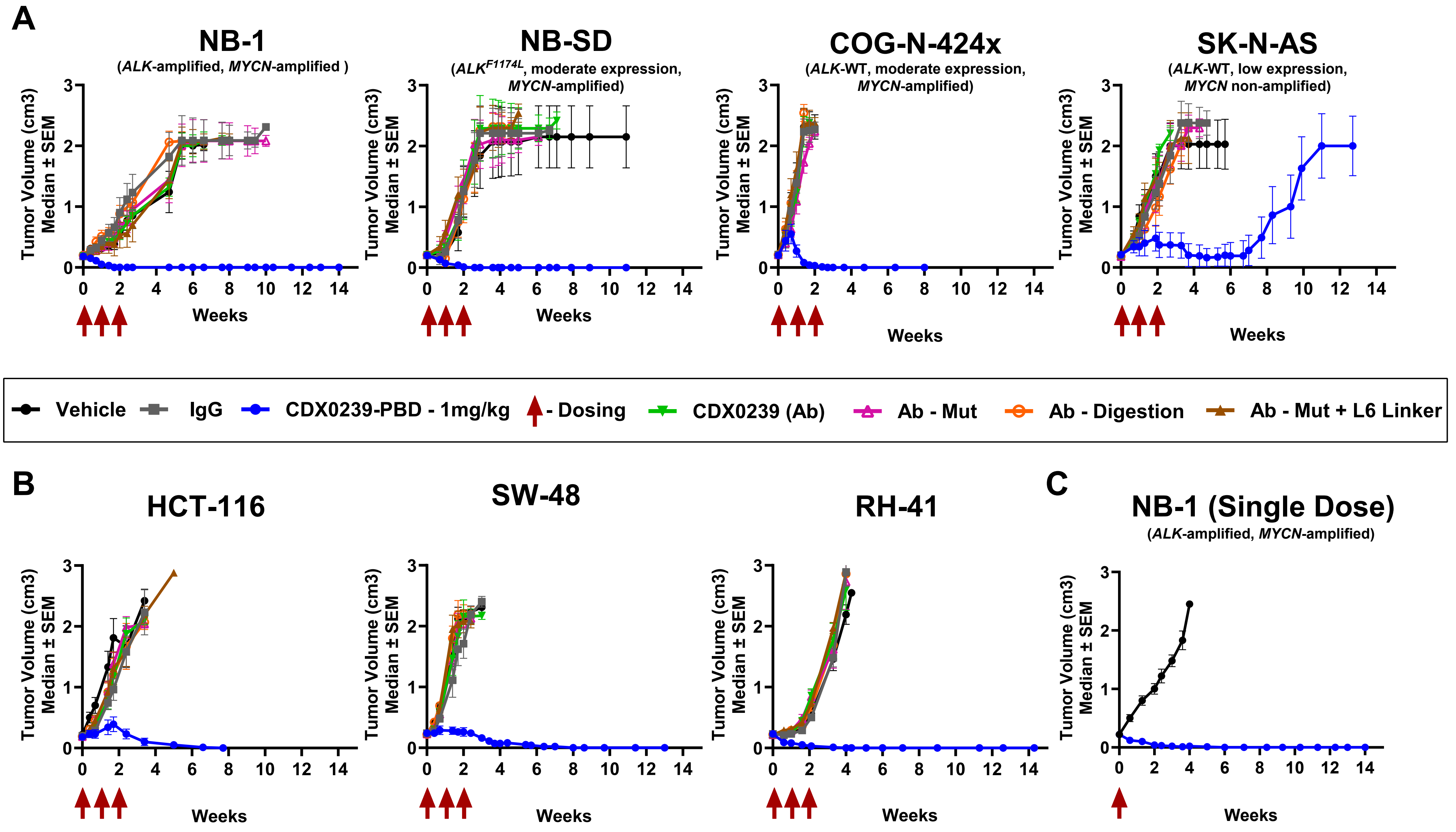

Supplemental Figure 4. CDX0239-PBD demonstrates potent *in vivo* antitumor activity in ALK-expressing xenograft models compared to multiple controls. (A) Average tumor volume in neuroblastoma xenograft models NB-1, NB-SD, COG-N-424x, and SK-N-AS and **(B)** average tumor volume colorectal carcinoma xenograft models HCT-116 and SW-48 and fusion-positive rhabdomyosarcoma xenograft model with 3 weekly treatments of 1 mg/kg CDX0239-PBD (blue line with circles) compared to IgG (grey line with squares), normal saline vehicle (black line with circles), CDX0239 (Ab, green line with triangles), CDX0239 with deglycosylation via asparagine to alanine mutation (Ab – Mut, pink line with triangles), CDX0239 with deglycosylation via enzymatic digestion (Ab – Digestion, orange line with circles), and CDX0239 with deglycosylation via asparagine to alanine mutation and with linker (Ab - Mut + L6 Linker, brown line with triangles). **(C)** Average tumor volume in neuroblastoma xenograft model NB-1 with a single 1 mg/kg dose of CDX0239-PBD (blue line with circles) compared to normal saline vehicle (black line with circles). All data are plotted as mean ± standard deviation with n=5 mice per treatment condition for all experiments, and each experiment was completed once. Exact p-values and datasets are located within the Source Data file. Source data are provided as a Source Data file.

Supplemental Figure 5

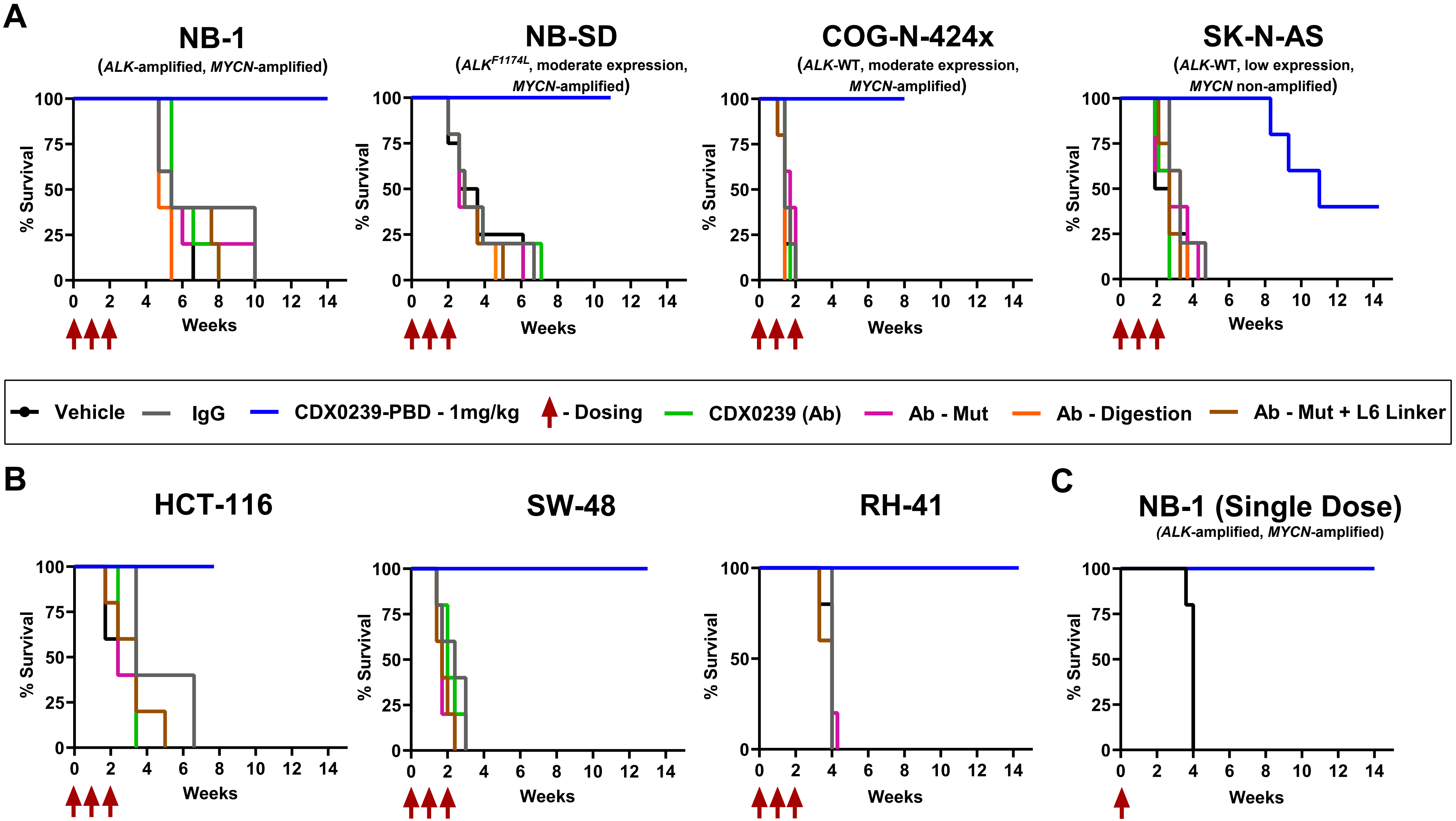

Supplemental Figure 5. Treatment with CDX0239-PBD leads to survival in ALK-expressing xenograft models compared to multiple controls. (A) Overall survival of neuroblastoma xenograft models NB-1, NB-SD, COG-N-424x, and SK-N-AS and **(B)** overall survival of colorectal carcinoma xenograft models HCT-116 and SW-48 and fusion-positive rhabdomyosarcoma xenograft model with 3 weekly treatments of 1 mg/kg CDX0239-PBD (blue line) compared to IgG (grey line), normal saline vehicle (black line), CDX0239 (Ab, green line), CDX0239 with deglycosylation via asparagine to alanine mutation (Ab – Mut, pink line), CDX0239 with deglycosylation via enzymatic digestion (Ab – Digestion, orange line), and CDX0239 with deglycosylation via asparagine to alanine mutation and with linker (Ab - Mut + L6 Linker, brown line). **(C)** Overall survival of neuroblastoma xenograft model NB-1 with a single 1 mg/kg dose of CDX0239-PBD (blue line) compared to normal saline vehicle (black line). Each treatment condition includes a sample size of n=5 mice and each experiment was completed once. Exact p-values and datasets are located within the Source Data file. Source data are provided as a Source Data file.

Supplemental Figure 6

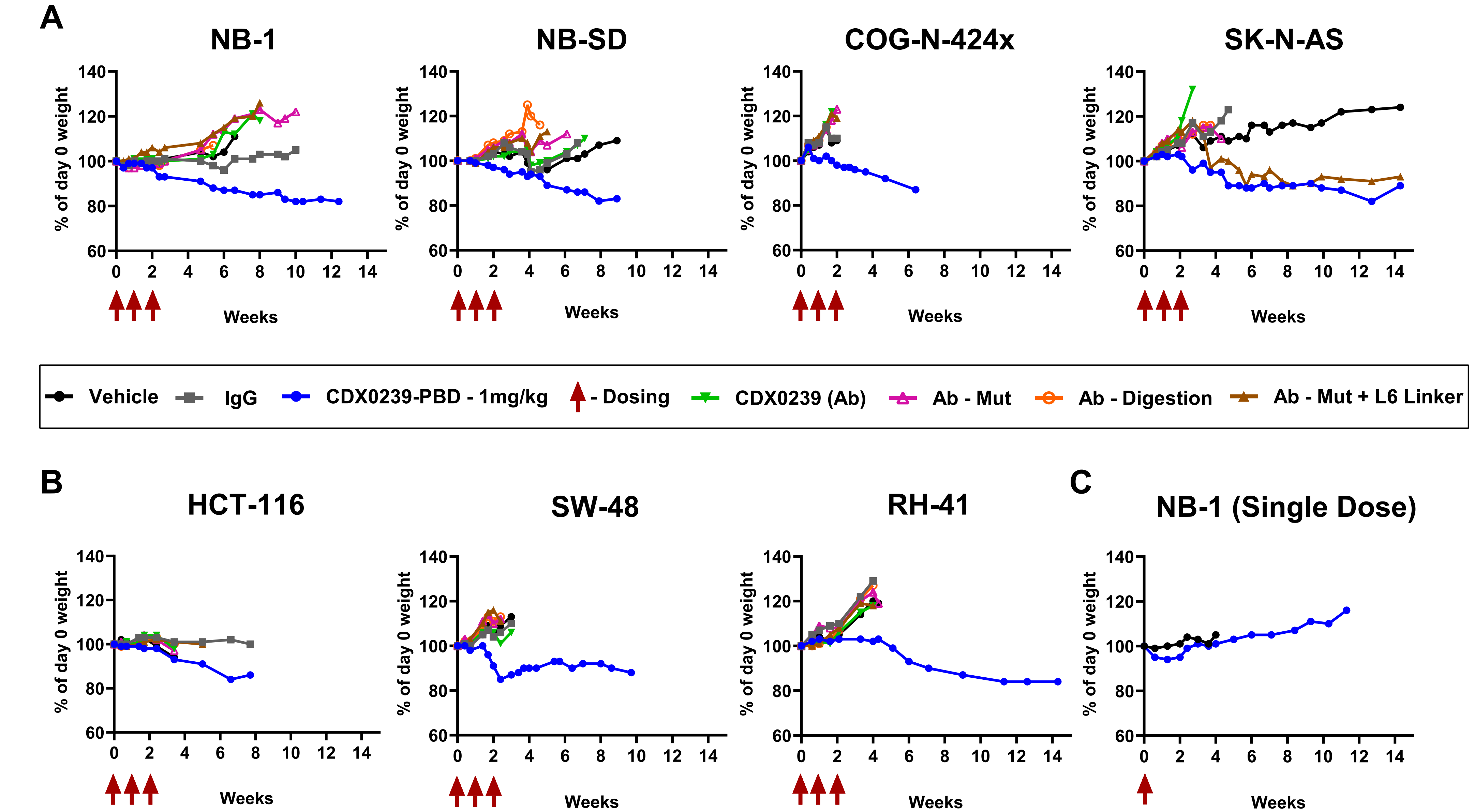

Supplemental Figure 6. Murine weights after treatment of CDX0239-PBD compared to multiple controls. (A) Murine weights of neuroblastoma xenograft models NB-1, NB-SD, COG-N-424x, and SK-N-AS and **(B)** murine weights of colorectal carcinoma xenograft models HCT-116 and SW-48 and fusion-positive rhabdomyosarcoma xenograft model with 3 weekly treatments of 1 mg/kg CDX0239-PBD (blue line with circles) compared to IgG (grey line with squares), normal saline vehicle (black line with circles), CDX0239 (Ab, green line with triangles), CDX0239 with deglycosylation via asparagine to alanine mutation (Ab – Mut, pink line with triangles), CDX0239 with deglycosylation via enzymatic digestion (Ab – Digestion, orange line with circles), and CDX0239 with deglycosylation via asparagine to alanine mutation and with linker (Ab - Mut + L6 Linker, brown line with triangles). **(C)** Murine weights of neuroblastoma xenograft model NB-1 with a single 1 mg/kg dose of CDX0239-PBD (blue line with circles) compared to normal saline vehicle (black line with circles). All data are plotted as mean weights from a total of n=5 mice per treatment condition for all experiments, and each experiment was completed once. Datasets are located within the Source Data file. Source data are provided as a Source Data file**.**

Supplemental Table 1

Neoplastic Histology	Mean TPM	Median TPM	SD TPM	N	Neoplastic Histology	Mean TPM	Median TPM	SD TPM	N
Neuroblastoma	37.78	21.64	102.52	201	Infantile Fibrosarcoma	1.11	1.11	0.11	2
Fusion + Rhabdomyosarcoma	23.55	17.69	20.96	49	Uterine Corpus Endometrioid Carcinoma	1.48	1.10	1.48	181
Dysembryoplastic Neuroepithelial Tumor	8.61	8.13	5.21	14	Sarcoma	2.80	1.10	8.96	61
Retinoblastoma†	7.55	7.55	1.46	2	Desmoplastic Small Round Cell Tumor	1.38	1.10	0.56	8
Ewing Sarcoma	9.78	6.75	9.27	85	Breast Invasive Carcinoma	1.20	1.09	0.53	1099
Ganglioglioma	5.33	2.62	7.98	47	Kidney Papillary Cell Carcinoma	1.47	1.08	2.39	289
Glioblastoma Multiforme	4.03	2.47	4.93	200	Kidney Clear Cell Carcinoma	1.18	1.08	0.32	532
Cutaneous Melanoma	6.91	2.27	38.75	469	Undifferentiated Pleomorphic Sarcoma	1.74	1.08	1.85	47
Diffuse Midline Glioma	3.94	2.25	6.62	82	Undifferentiated Hepatic Sarcoma	1.08	1.08	0.11	2
High-Grade Glioma/Astrocytoma	2.95	0.72	6.18	149	Thymoma	1.15	1.07	0.29	119
Melanoma	6.74	6.36	3.56	7	Dedifferentiated Liposarcoma	1.29	1.06	0.85	50
Embryonal Tumor with Multilayered Rosettes	5.73	5.73	6.26	2	Adrenocortical Carcinoma	1.45	1.06	2.90	96
Spindle Cell/Sclerosing Rhabdomyosarcoma	5.51	3.43	5.87	5	Prostate Adenocarcinoma	1.07	1.05	0.075	496
Uveal Melanoma	5.42	3.18	6.36	79	Esophageal Carcinoma	1.10	1.05	0.14	182
Pheochromocytoma & Paraganglioma	4.80	2.76	6.31	184	Undifferentiated Sarcoma Nos	1.09	1.05	0.09	13
Neurofibroma	2.74	2.74	1.29	2	Lymphoma	1.09	1.05	0.10	50
Desmoplastic Infantile Astrocytoma and Ganglioglioma	5	2.73	4.98	3	Gastrointestinal Stromal Tumor	1.25	1.05	0.35	7
Synovial Sarcoma	3.47	2.49	2.46	41	Cervical & Endocervical Cancer	1.12	1.04	0.28	306
Germ Cell Tumor	2.35	2.35	0.49	2	Stomach Adenocarcinoma	1.10	1.04	0.25	414
Ependymoma	2.43	2.23	1.27	102	Head & Neck Squamous Cell Carcinoma	1.09	1.04	0.21	520
Supratentorial Embryonal Tumor Nos	6.10	2.19	8.28	19	Osteosarcoma	1.11	1.04	0.28	189
Diffuse Intrinsic Pontine Glioma	2.22	2.15	1.24	9	Epithelioid Sarcoma	1.04	1.04	0.01	3
Thyroid Carcinoma	4.79	1.65	59.15	516	Undifferentiated Spindle Cell Sarcoma	1.04	1.04	0.01	2
Malignant Peripheral Nerve Sheath Tumor	4.73	1.61	5.70	9	Leiomyosarcoma	10.99	1.03	84.43	78
Fusion - Rhabdomyosarcoma	2.99	1.53	4.64	61	Diffuse Large B-Cell Lymphoma	1.16	1.03	0.61	47
Ovarian Serous Cystadenocarcinoma	2.25	1.50	2.16	427	Kidney Chromophobe	1.10	1.03	0.23	66
Myofibromatosis	1.48	1.48	0.68	2	Hepatoblastoma	1.35	1.03	0.94	19
Nasopharyngeal Carcinoma	5.26	1.44	6.97	3	Bladder Urothelial Carcinoma	1.33	1.02	4.34	407
Choroid Plexus Carcinoma	2.33	1.39	2.85	26	Cholangiocarcinoma	1.06	1.02	0.08	37
Uterine Carcinosarcoma	1.92	1.36	2.37	57	Rectum Adenocarcinoma	1.07	1.02	0.34	93
Thymic Carcinoma	1.33	1.33	0.36	2	Acute Lymphoblastic Leukemia	1.09	1.02	0.40	878
Meningioma	1.26	1.30	0.10	3	Ini-Deficient Soft Tissue Sarcoma Nos	1.02	1.02	0.03	2
Medulloblastoma	2.39	1.29	3.60	125	Fibrolamellar Hepatocellular Carcinoma	1.03	1.02	0.05	8
Mesothelioma	2.01	1.25	2.70	87	Acute Myeloid Leukemia	1.10	1.01	0.22	538
Fibromatosis	1.24	1.24	0.18	4	Hepatocellular Carcinoma	1.02	1.01	0.05	375
Low-Grade Glioma/Astrocytoma	1.88	1.23	2.08	86	Colon Adenocarcinoma	1.04	1.01	0.10	293
Atypical Teratoid/Rhabdoid Tumor	1.49	1.22	0.69	4	Inflammatory Myofibroblastic Tumor	1.08	1.01	0.14	4
Testicular Germ Cell Tumor	1.31	1.17	0.53	155	Acute Megakaryoblastic Leukemia	1.04	1.01	0.10	93
Wilms Tumor	1.45	1.16	0.85	137	Acute Leukemia Of Ambiguous Lineage	1.01	1.01	0.01	2
Lung Squamous Cell Carcinoma	1.42	1.16	1.02	498	Leukemia, Unspecified Subtype	1.01	1.01	0.01	2
Lung Adenocarcinoma	1.38	1.15	1.38	516	Myeloproliferative Neoplasm	1.01	1.01	0.01	2
Rhabdoid Tumor	1.15	1.15	0.156	2	Juvenile Myelomonocytic Leukemia	1.01	1.00	0.03	4
Myxofibrosarcoma	1.71	1.14	1.58	17	Acute Lymphoblastic Leukemia	1.00	1.00	0.01	10
Myoepithelial Carcinoma	1.13	1.14	0.01	3	Oligodendroglioma	0.62	0.74	0.34	3
Pancreatic Adenocarcinoma	1.52	1.11	4.53	179	Small Cell Lung Cancer	0.81	0.09	1.7	79

Supplemental Table 1. *ALK* RNA expression in neoplastic histologies. *ALK* RNA expression quantified as the mean and median with standard deviation (SD) values of transcripts per million (TPM) with total number of samples (N) included in analysis. Retinoblastoma† was excluded from outlier analysis of *ALK* RNA expression due to small sample size of n=2.

Supplemental Table 2

Normal Histology	Mean TPM	Median TPM	SD TPM	n	Normal Histology	Mean TPM	Median TPM	SD TPM	n
Pituitary	4.22	3.53	2.88	283	Prostate	0.12	0.09	0.12	245
Testis	3.16	3.08	1.34	361	Skin	0.10	0.08	0.07	1305
Colon	1.17	0.82	1.18	779	Uterus	0.12	0.08	0.14	142
Brain	0.95	0.76	0.85	2642	Lung	0.11	0.08	0.12	578
Nerve	0.62	0.47	0.66	619	Fallopian Tube	0.10	0.08	0.05	9
Esophagus	0.54	0.41	0.54	1445	Vagina	0.17	0.07	0.4	156
Salivary Gland	0.20	0.18	0.13	162	Stomach	0.24	0.07	0.36	359
Small Intestine	0.49	0.18	0.85	187	Muscle	0.07	0.04	0.09	803
Breast	0.23	0.14	0.34	459	Artery	0.06	0.04	0.11	1335
Bladder	0.18	0.13	0.14	21	Heart	0.04	0.03	0.04	861
Breast	0.23	0.13	0.34	459	Spleen	0.04	0.03	0.03	241
Cervix	0.26	0.12	0.33	19	Kidney	0.05	0.02	0.10	89
Adrenal Gland	0.21	0.11	0.33	258	Pancreas	0.02	0.02	0.02	328
Adipose	0.18	0.11	0.22	1204	Cells	0.02	0.02	0.02	678
Thyroid	0.12	0.11	0.12	653	Liver	0.02	0.01	0.03	226
Ovary	0.13	0.10	0.10	180	Blood	0.01	0.01	0.01	755

Supplemental Table 2. *ALK* RNA expression in normal pediatric histologies. *ALK* RNA expression quantified as the mean and median with standard deviation (SD) values of transcripts per million (TPM) with total number of samples (n) included in analysis.

Supplemental Table 3

	A	B	C	D	E	F	G	H	I	J	K	L
1	Placenta	Adrenal cortex	Adrenal cortex	Adrenal medulla	Adrenal medulla	Tonsil	Tonsil	NB –post-chemotherapy	NB –post-chemotherapy	NB – post-chemotherapy	NB – post-chemotherapy	NB – post-chemotherapy
2	NB – post-chemotherapy	NB – post-chemotherapy	NB – diagnostic	NB – diagnostic	NB – diagnostic	NB – diagnostic	Placenta	NB – post-chemotherapy	NB – post-chemotherapy	NB – diagnostic	NB – diagnostic	NB – diagnostic
3	NB – relapsed	NB – relapsed	NB – post-chemotherapy	NB – post-chemotherapy	NB – post-chemotherapy	NB – post-chemotherapy	Placenta	NB – diagnostic	NB – diagnostic	NB – relapsed	NB – relapsed	NB – post-chemotherapy
4	NB – post-chemotherapy	NB – post-chemotherapy	NB – post-chemotherapy	NB – post-chemotherapy	NB – relapsed	NB – relapsed	Placenta	NB – diagnostic	NB – diagnostic	NB –post-chemotherapy	NB –post-chemotherapy	Ganglio-neuroblastoma
5	NB – diagnostic	NB – diagnostic	NB – relapsed	NB – relapsed	NB – diagnostic	NB – diagnostic	Placenta	Ganglio-neuroblastoma	Ganglio-neuroblastoma	Ganglio-neuroblastoma	Ganglio-neuroblastoma	NB – post-chemotherapy
6	NB – post-chemotherapy	NB – post-chemotherapy	NB – diagnostic	NB – diagnostic	NB – diagnostic	NB – diagnostic	Placenta	NB – diagnostic	NB – diagnostic	NB – post-chemotherapy	NB – post-chemotherapy	NB – diagnostic
7	NB – post-chemotherapy	NB – post-chemotherapy	Ganglio-neuroblastoma	Ganglio-neuroblastoma	NB – diagnostic	NB – diagnostic	Placenta	NB – diagnostic	NB – diagnostic	Ganglioneuroma	Ganglioneuroma	NB – post-chemotherapy
8	NB – post-chemotherapy	NB – post-chemotherapy	NB – post-chemotherapy	NB – post-chemotherapy	Ganglio-neuroblastoma	Ganglio-neuroblastoma	Placenta	NB – diagnostic	NB – diagnostic	NB – diagnostic	NB – diagnostic	NB – diagnostic
9	NB – post-chemotherapy	NB – post-chemotherapy	NB – diagnostic	NB – diagnostic	NB – post-chemotherapy	NB – post-chemotherapy	Placenta	NB – post-chemotherapy	NB – post-chemotherapy	NB – diagnostic	NB – diagnostic	NB – diagnostic
10	NB – diagnostic	NB – diagnostic	NB – diagnostic	NB – diagnostic	NB – diagnostic	NB – diagnostic	Placenta	NB – diagnostic	NB – diagnostic	NB – relapsed	NB – relapsed	NB – diagnostic
11	NB – diagnostic	NB – diagnostic	NB – post-chemotherapy	NB – post-chemotherapy	NB – diagnostic	NB – diagnostic	Placenta	NB – diagnostic	NB – diagnostic	NB – diagnostic	NB – diagnostic	NB – diagnostic
12	NB – relapsed	NB – relapsed	NB – diagnostic	NB – diagnostic	Placenta	Placenta	Placenta	Placenta	Placenta	Placenta	Placenta	Placenta

Supplemental Table 3. Legend for neuroblastoma tumor tissue microarray (TMA). Tumor samples and healthy tissues were stained for ALK (D5F3). All neuroblastoma tumors are displayed as adjacent duplicate cores.

Supplemental Table 4

	A	B	C	D	E	F	G	H	I	J	K	L	M
1	Placenta	Adrenal Cortex	Adrenal Cortex	Adrenal Medulla	Adrenal Medulla	Aorta	Aorta	Artery	Artery	Bladder	Bladder	Bone marrow	Bone marrow
2	Brain (cerebellum)	Brain (cerebellum)	Brain (cortex)	Brain, (cortex)	Breast	Breast	Placenta	Cervix Uteri	Cervix Uteri	Choroid plexus	Choroid plexus	Esophagus	Esophagus
3	Cornea	Cornea	Fallopian Tube	Fallopian Tube	Gallbladder	Gallbladder	Placenta	Epiphyseal plate	Epiphyseal plate	Heart	Heart	Kidney (cortex)	Kidney (cortex)
4	Kidney (medulla)	Kidney (medulla)	Colon	Colon	Liver	Liver	Placenta	Lung	Lung	Lymph Node	Lymph Node	Ovary	Ovary
5	Pancreas	Pancreas	Parathyroid	Parathyroid	Pituitary	Pituitary	Placenta	Prostate	Prostate	Salivary Gland	Salivary Gland	Skeletal Muscle	Skeletal Muscle
6	Skin	Skin	Small Intestine	Small Intestine	Smooth muscle	Smooth muscle	Placenta	Spinal Cord	Spinal Cord	Spleen	Spleen	Stomach	Stomach
7	Testis	Testis	Thymus	Thymus	Thyroid Gland	Thyroid Gland	Placenta	Tonsil	Tonsil	Ureter	Ureter	Uterus	Uterus

Supplemental Table 4. Legend for normal tissue microarray (TMA). Tissue samples were stained for ALK (D5F3) and displayed as adjacent duplicate cores from each tissue unless listed as a single sample.

	IgG Geometric Mean	ALK Geometric Mean	ALK:IgG Geometric Mean
NB-1	157	104,993	668.7
COG-N-424x	62.2	1,721	27.7
NB-SD	26.2	391	15.0
SK-N-AS	236	835	3.5
NGP *ALK* WT	711	6104	8.6
NGP *ALK* KO	587	912	1.6
RH-41	751	5,275	7.02
SW-48	1,582	15,446	9.76
HCT-116	304	1,699	5.59

Supplemental Table 5. Flow cytometry histogram geometric means. ALK and IgG geometric means with corresponding ALK:IgG geometric mean ratios in xenograft models, and in neuroblastoma NGP *ALK* wild-type (WT) and NGP *ALK* knockout (KO) cell lines.

Neuroblastoma Cell Line	CDX0239-PBD IC_{50} (pM)	Free PBD IC_{50} (pM)
NB-1	16.1	342
NB-SD	22.5	619
SK-N-AS	5,070	64.3
NGP ALK WT	42.79	31.08
NGP ALK KO	24,400,000	32.73

Supplemental Table 6. IC_{50} values of CDX0239-PBD and free PBD in neuroblastoma cell lines.

Supplemental Table 7

Model	Disease	*ALK* Status	*MYCN* Status	*TP53* Status	CDX0239-PBD Dose (mg/kg)	n	Response	Tumor Size p-value vs. Vehicle	**Median KM (days)**	**KM p-value vs. Vehicle**
NB-1	Neuroblastoma	WT; Amplified	Amplified	WT	1	5	Complete Response	<0.0001	>90	0.0017
NB-SD	Neuroblastoma	c.3522C>A:F1174L	Amplified	c.527G>T_p.C176F	1	5	Complete Response	0.0015	>70	0.0102
COG-N-424x	Neuroblastoma	WT	Amplified	WT	1	5	Complete Response	<0.0001	>50	0.0018
SK-N-AS	Neuroblastoma	WT	Non-Amplified	c.503A>G_p.H168R	1	5	Partial Response	0.0008	56	0.0463
NGP *ALK* WT	Neuroblastoma	WT	Amplified	c.432C>G_p.R114G	1	5	Complete Response	<0.0001	>100	0.0019
NGP *ALK* KO	Neuroblastoma	KO	Amplified	c.432C>G_p.R114G	1	5	Progressive Disease	0.3065	32	0.0583
HCT-116	Colorectal Carcinoma	WT	Not applicable	WT	1	5	Complete Response	<0.0001	>40	0.0003
SW-48	Colorectal Carcinoma	WT	Not applicable	WT	1	5	Complete Response	<0.0001	>80	0.0023
RH-41	Fusion-positive Rhabdomyosarcoma (*PAX3::FOXO1* fusion)	WT	Not applicable	WT	1	5	Complete Response	<0.0001	>90	0.0012

Supplemental Table 7. Xenograft model genomic, treatment, and response data. Xenografts used for *in vivo* studies listed with associated *ALK* status, *MYCN* status, *TP53* status, CDX0239-PBD dose in mg/kg, number of murine models enrolled per condition (n), tumor volume response, p-value for tumor size in CDX0239-PBD treatment group vs. normal saline vehicle treatment group, median Kaplan-Meier survival estimate (KM) in days, and p-value for median KM logrank in CDX0239-PBD treatment group vs normal saline vehicle treatment group.
