# Supplementary material for: A humanized anaplastic lymphoma kinase (ALK)-directed antibody-drug conjugate with pyrrolobenzodiazepine payload demonstrates efficacy in ALK-expressing cancers
Source: Nat Commun. 2025 Aug 15;16:7578. doi: 10.1038/s41467-025-62979-1 (PMC12354906; doi:10.1038/s41467-025-62979-1)
Supplement: Supplementary file 2 — Reporting Summary [file 41467_2025_62979_MOESM2_ESM.pdf]

nature portfolio

Corresponding author(s): Dr. Yael P Mossé

Last updated by author(s): 7/6/2025

Reporting Summary

Nature Portfolio wishes to improve the reproducibility of the work that we publish. This form provides structure for consistency and transparency in reporting. For further information on Nature Portfolio policies, see our Editorial Policies and the Editorial Policy Checklist.

Statistics

For all statistical analyses, confirm that the following items are present in the figure legend, table legend, main text, or Methods section.

n/a	Confirmed	
☐	☒	The exact sample size (n) for each experimental group/condition, given as a discrete number and unit of measurement
☐	☒	A statement on whether measurements were taken from distinct samples or whether the same sample was measured repeatedly
☐	☒	The statistical test(s) used AND whether they are one- or two-sided<br>*Only common tests should be described solely by name; describe more complex techniques in the Methods section.*
☐	☒	A description of all covariates tested
☐	☒	A description of any assumptions or corrections, such as tests of normality and adjustment for multiple comparisons
☐	☒	A full description of the statistical parameters including central tendency (e.g. means) or other basic estimates (e.g. regression coefficient) AND variation (e.g. standard deviation) or associated estimates of uncertainty (e.g. confidence intervals)
☒	☐	For null hypothesis testing, the test statistic (e.g. F, t, r) with confidence intervals, effect sizes, degrees of freedom and P value noted<br>*Give P values as exact values whenever suitable.*
☒	☐	For Bayesian analysis, information on the choice of priors and Markov chain Monte Carlo settings
☒	☐	For hierarchical and complex designs, identification of the appropriate level for tests and full reporting of outcomes
☒	☐	Estimates of effect sizes (e.g. Cohen's d, Pearson's r), indicating how they were calculated

Our web collection on statistics for biologists contains articles on many of the points above.

Software and code

Policy information about availability of computer code

Data collection	Links to the publicly datasets used for ALK RNA analysis are included in the 'Data' section below.
Data analysis	A polyA selection method was used to quantify ALK RNA expression data as log2(transcripts per million) from publicly available data from the Tumor Compendium v11 of UCSC's Treehouse Childhood Cancer Initiative and the Genotype-Tissue Expression Project. Boxplots were generated in R version 4.2.3 using ggplot2 function from Tidyverse package version 2.0.0. Code scripts are available at the Github repository marislab/ALK_ADC v1.0.1 and is archieved via Zenodo (https://doi.org/10.5281/zenodo.15830580). The repository includes the code used to determine histologies that were outliers of ALK RNA expression to generate Figures 1 A-B in this manuscript.

For manuscripts utilizing custom algorithms or software that are central to the research but not yet described in published literature, software must be made available to editors and reviewers. We strongly encourage code deposition in a community repository (e.g. GitHub). See the Nature Portfolio guidelines for submitting code & software for further information.

Data

Policy information about availability of data

All manuscripts must include a data availability statement. This statement should provide the following information, where applicable:

- Accession codes, unique identifiers, or web links for publicly available datasets
- A description of any restrictions on data availability
- For clinical datasets or third party data, please ensure that the statement adheres to our policy

Source data are provided with this paper. The datasets generated during and/or analyzed during the current study are available in the main text, supplementary information, or source data provided with this manuscript. Individual histology slides and pictographs used in this study are available from the corresponding author on request. All biologic material other than CDX0239 and CDX0239-PBD is available upon request due to intellectual property restrictions. Cell lines and xenografts may be made available upon request pending resource restriction. The links for the publicly available datasets from the Tumor Compendium v11 of UCSC Treehouse Childhood Cancer Initiative and from The Genotype-Tissue Expression Project v8 used for ALK RNA expression analysis to generate Figure 1, Supplemental Table 1, and Supplemental Table 2 are provided below.

UCSC Treehouse Childhood Cancer Initiative Tumor Compendium v11:
The Tumor Compendium v11 is available from the UCSC Treehouse Childhood Cancer Initiative portal at https://treehousegenomics.soe.ucsc.edu/public-data/. Direct download and access instructions are provided on the portal. No restrictions apply.

The Genotype-Tissue Expression (GTEx) Project v8:
The GTEx data set is available from the GTEx Portal at https://gtexportal.org/home/datasets. All processed RNA sequencing data are publicly available and raw data may require dbGap authorization (Accession: phs000424.v8.p2).

Research involving human participants, their data, or biological material

Policy information about studies with human participants or human data. See also policy information about sex, gender (identity/presentation), and sexual orientation and race, ethnicity and racism.

Reporting on sex and gender	Samples for neuroblastoma tumor core and healthy tissue TMA production and analysis were de-identified with only donor sex and age information available.
Reporting on race, ethnicity, or other socially relevant groupings	All histology slides used for human neuroblastoma and normal tissue analyses were de-identified from race, ethnicitiy, or other socially relevant groupings.
Population characteristics	All histology slides used for human neuroblastoma and normal tissue analyses were de-identified from population characteristics.
Recruitment	There was no additional recruitment of human participants, data, or biological material for this study as patient microarrays were completed prior to this study.
Ethics oversight	Patient samples obtained for TMA creation and annotation with clinical co-variate data was approved by the Children's Hospital of Philadelphia Institutional Review Board Protocol 18-015545, and the study was conducted in accordance with the United States Common Rule. Neuroblastoma tumors cores used to produce the TMAs and samples used to produce PDX models were collected after written, signed and dated informed consent provided by the patient parents/guardians. Tumor cores used to produce the TMAs were biobanked at the Children's Hospital of Philadelphia and no additional compensation was provided. Healthy donors provided informed consent through the University of Pennsylvania Immunology Core.

Note that full information on the approval of the study protocol must also be provided in the manuscript.

Field-specific reporting

Please select the one below that is the best fit for your research. If you are not sure, read the appropriate sections before making your selection.

[x] Life sciences   [ ] Behavioural & social sciences   [ ] Ecological, evolutionary & environmental sciences

For a reference copy of the document with all sections, see nature.com/documents/nr-reporting-summary-flat.pdf

Life sciences study design

All studies must disclose on these points even when the disclosure is negative.

Sample size	All in vitro experiments were completed with at least three technical replicates to ensure adequate statistical analyses, and all attempts at replication were successful. Neuroblastoma tumor cores analyzed for ALK immunohistochemistry staining for H-score analysis included a sample size of 55 tumors, with duplicate tumor cores provided for 48 tumors, triplicate tumor cores provided for 1 tumor, and a single tumor core provided for 6 tumors. Average H-scores between cores from the same tumor were used for analysis when duplicate or triplicate cores were provided. A sample size of 43 normal tissues with 4 samples per tissue were used for normal tissue ALK immunhistochemistry staining. In

	vivo studies included sample sizes of 5 mice per xenograft treatment group as per our previous in vivo studies to ensure adequate statistical power.
Data exclusions	No data was excluded in the analyses of generated data of this research.
Replication	All in vitro were repeated a minimum of three times for biological reproducibility. In vivo studies were completed as a single replicate per treatment xenograft treatment group given resource limitation for repeat in vivo studies.
Randomization	Each animal was randomized into each treatment group for a total sample size of 5 mice per randomized treatment group.
Blinding	Investigators were not blinded to any in vitro experiments. Investigators were not blind to the randomization of the in vivo studies to complete continuous tumor volume, mouse weight, and survival analysis. Blinding was not possible given the same authors treated the animals and measured tumor volumes of the corresponding animals.

Reporting for specific materials, systems and methods

We require information from authors about some types of materials, experimental systems and methods used in many studies. Here, indicate whether each material, system or method listed is relevant to your study. If you are not sure if a list item applies to your research, read the appropriate section before selecting a response.

Materials & experimental systems

n/a	Involved in the study
☐	☒ Antibodies
☐	☒ Eukaryotic cell lines
☒	☐ Palaeontology and archaeology
☐	☒ Animals and other organisms
☐	☒ Clinical data
☒	☐ Dual use research of concern
☒	☐ Plants

Methods

n/a	Involved in the study
☒	☐ ChIP-seq
☐	☒ Flow cytometry
☒	☐ MRI-based neuroimaging

Antibodies

Antibodies used	Murine anti-ALK CDX0239 (generated and validated with this research as per methods section), rabbit anti-ALK total D5F3 (Cell Signaling, #3633S, Lot #13), rabbit anti-phosphorylted ALK (Tyr1604, Cell Signaling, #3341L, Lot #7), rabbit anti-phosphorylated H2AX (Ser139, Cell Signaling, #2577S, Lot #14), rabbit anti-cleaved caspase-3 (Asp175, Cell Signaling, #9664L, Lot #22), rabbit anti-PARP (Cell signaling, #9532S, Lot #10), rabbit anti-beta actin (Cell Signaling, #4967S, Lot #10), rabbit anti-phospho-B-Raf (Cell Signaling, #2696S, Lot #3), goat anti-rabbit IgG HRP-conjugated secondary antibody (Cell Signaling, #7074S). All antibodies utilized for western blots were used at a dilution of 1:1,000 other than goat anti-rabbit IgG HRP-conjugated secondary antibody which was used at a dilution of 1:5,000. Anti-ALK total D5F3 used at a dilution of 1:500 for immunohistochemistry studies.
Validation	Rabbit anti-ALK D5F3 is FDA approved and CLIA-certified for ALK staining for human tissue samples. Murine anti-ALK CDX0239 affinity was determined with bio-layer interferometry with confirmed human and mouse cross-species reactivity with flow cytometry data included in this manuscript. Rabbit anti-ALK total #3333S was validated for species reactivity by Cell Signaling via a minimum of one approved application (e.g Western Blot). Rabbit anti-phosphorylated ALK #3341L was validated for species reactivity by Cell Signaling via a minimum of one approved application (e.g Western Blot). Rabbit anti-phosphorylated H2A.X #2577S was validated for species reactivity by Cell Signaling via a minimum of one approved application (e.g Western Blot). Rabbit ant-cleaved caspase 3 #9664L was validated for species reactivity by Cell Signaling via a minimum of one approved application (e.g Western Blot). Rabbit anti-PARP #9532S was validated for species reactivity by Cell Signaling via a minimum of one approved application (e.g Western Blot). Rabbit anti-beta actin #4790L was validated for species reactivity by Cell Signaling via a minimum of one approved application (e.g Western Blot). Rabbit anti-phospho-B-Raf #2696S was validated for species reactivity by Cell Signaling via a minimum of one approved application (e.g. Western Blot). Goat anti-rabbit IgG HRP-conjugated secondary antibody #7074S was validated for species reactivity by Cell Signaling via a minimum of one approved application (e.g Western Blot). All validation methods are available on the Cell Signaling manufacturer's website (cellsignal.com).

Eukaryotic cell lines

Policy information about cell lines and Sex and Gender in Research

Cell line source(s)	Cell lines were obtained from the Children's Hospital of Philadelphia (CHOP) cell line bank.<br>NB-1: human cell line derived from 27 month old male neuroblastoma patient from from a metastatic lymph node lesion<br>NB-SD: human cell line derived from a sex and age unspecified neuroblastoma patient from a metastatic bone marrow lesion<br>IMR-32: human cell line derived from a 13 month old male from an abdominal mass biopsy<br>NGP: human cell line derived from a 30 month old male neuroblastoma patient (unknown primary site)<br>NGP knockout (KO): human cell line derived from a 30 month old male neuroblastoma patient (unknown primary site), knockout generated via CRISPR knockout with lentiCRISPR v2<br>SK-N-AS: human cell line derived from an 8 year old female neuroblastoma patient from a metastatic bone marrow lesion

Authentication	Short tandem repeat (STR) testing was completed on each cell line for authentication.
Mycoplasma contamination	All cell lines used in this study were confirmed to be negative for mycoplasma contamination
Commonly misidentified lines (See ICLAC register)	n/a

Animals and other research organisms

Policy information about studies involving animals; ARRIVE guidelines recommended for reporting animal research, and Sex and Gender in Research

Laboratory animals	Female CB17 SCID mice procured from Charles River and housed in female mice-only Supermouse 750 cages at 5 mice per cage with corncob bedding, with light cycles starting at 0600 and dark cycles starting at 1800. Temperature was maintained between 20-26 oC and humidity was maintained between 30-70%. Mice were implanted at an age of 6-8 weeks for all animal studies in this research. All animal studies were approved by the Children's Hospital of Philadelphia Institutional Animal Care and Use Committee protocol no. IAC 000643 with adherence to the National Institutes of Health guide for Care and Use of Laboratory Animals accredited by the Association for Assessment and Accreditation of Laboratory Animal Care (AAALAC).
Wild animals	n/a
Reporting on sex	Female mice only were used for all animal studies to ensure appropriate animal housing conditions.
Field-collected samples	n/a
Ethics oversight	All studies were approved by the Children's Hospital of Philadelphia Institutional Animal Care and Use Committee protocol no. IAC 000643.

Note that full information on the approval of the study protocol must also be provided in the manuscript.

Plants

Seed stocks	n/a
Novel plant genotypes	n/a
Authentication	n/a
